# Autism Transition to Adulthood Groups (ATAG): protocol for a feasibility RCT of a new peer-group intervention to promote successful transition to adulthood for autistic young people

**DOI:** 10.1136/bmjopen-2024-089802

**Published:** 2024-11-19

**Authors:** Kate Cooper, Annabel Burnley, Leon Allain, Bryony Beresford, Laura Crane, Maximiliano Vazquez Morales, Lucy Portway, Benjamin Redmayne, Ailsa Russell, W Mandy

**Affiliations:** 1 Research Department of Clinical, Educational, & Health Psychology, University College London, London, UK; 2 Centre for Applied Autism Research, Department of Psychology, University of Bath, Bath, UK; 3 Ambitious about Autism, London, UK; 4 Social Policy Research Unit, University of York, York, UK; 5 University College London, London, UK; 6 Department of Disability, Inclusion and Special Needs, University of Birmingham, Birmingham, UK; 7 Population Health Sciences, University of Bristol, Bristol, UK; 8 University of Bristol, Bristol, UK

**Keywords:** Feasibility Studies, Internet, MENTAL HEALTH, Quality of Life, Social Support

## Abstract

**Introduction:**

Autism is a lifelong neurodevelopmental condition diagnosed on the basis of differences in social communication, interaction and repetitive behaviours, including sensory sensitivities. Autistic individuals without intellectual disabilities often face barriers to positive adult outcomes and are at high risk for poor health, including mental health issues, which could be mitigated by improving well-being. Young people should receive support to increase their well-being during the transition to adulthood, when social and family support often reduces. This is the protocol for a feasibility randomised controlled trial (RCT) of an online peer-group intervention, ‘Understanding You, Discovering You’ (UYDY). Objectives included assessing recruitment and retention rates, acceptability of procedures, characterising usual care, assessing the acceptability of UYDY and care as usual (CAU) and calculating outcome measure variances for a full trial.

**Methods and analysis:**

This two-arm parallel feasibility RCT includes a nested qualitative evaluation. Seventy participants aged 16–25 years old with a clinical autism diagnosis will be randomised on a 1:1 basis to UYDY or CAU. Exclusion criteria include risk of harm to self or others, receipt of postdiagnostic support in the past 12 months, and literacy levels such that the written session materials are not accessible. UYDY, a 6-week online peer-group intervention, will cover topics such as understanding autism, problem-solving and accessing services, and is facilitated by an autistic person and social care professionals. The main outcomes from the feasibility trial will be collecting data on (1) recruitment and retention rates; (2) the acceptability of randomisation and outcome measurement procedures; (3) CAU accessed by participants; (4) acceptability of the interventions; (5) clinical outcome measure variances (see below). See the Statistical methods section below for how this will be assessed in the current study. Clinical outcomes will be measured at baseline, and 8, 16 and 24 weeks post-randomisation. The primary clinical outcome is well-being, assessed using the Warwick Edinburgh Mental Wellbeing Scale. Secondary clinical outcomes include autism social identification, quality of life, social support and loneliness. Adverse events will be monitored and reported. Carer impact will also be measured. Participants will be recruited from England and Wales via charities and NHS services. Qualitative interviews will be conducted to explore the acceptability of trial participation including randomisation and the interventions.

**Ethics and dissemination:**

Ethical approval has been obtained from the HRA and NHS REC (23/WA/0113). Informed consent will be collected from all participants (see online supplemental material for an example consent form). Results will inform the design of a full RCT and will be disseminated through peer-reviewed journals, conferences, and stakeholder events.

**Trial registration number:**

ISRCTN10513626.

**STRENGTHS AND LIMITATIONS OF THIS STUDY**

- Feasibility randomised controlled trial of a new intervention for autistic 16–25-year olds.
- Coproduced intervention and trial design.
- Nested qualitative evaluation will investigate lived experiences of the trial and interventions.
- It will not be possible to make claims about the effectiveness of the intervention from this feasibility study.

## Introduction

 Autism is a lifelong neurodevelopmental condition diagnosed on the basis of differences in social communication and interaction and restricted and repetitive behaviours, including sensory sensitivities.^1^ In this paper, we focus on autistic young people without intellectual disability. Autistic individuals experience many barriers to good adult outcomes and are at risk of poor health^2^ and social outcomes.^3^ This includes being at high risk for mental health problems,^4^ which are more common in those aged 16–24 years as compared with those aged 0–15 years.^5^ It is important to research ways to reduce risk factors at this life stage. Moreover, improving well-being has been shown to reduce severity of mental health problems and prevent their onset,^6 7^ so well-being is a promising treatment target in all groups, and especially populations at increased risk for mental health problems.

The transition to adulthood is a moment of opportunity but also one of risk, when existing supports and structures are lost at a time of increasingly complex demands. In this paper, we define the transition to adulthood as applying to young people aged 16–25 years, which is in line with service provision for young adults in the UK, however, there is debate about the definition of adolescence, youth and young adulthood, and the boundaries between these life stages in both the general population^8^ and autistic individuals specifically.^9^ This life stage sees a reduction of formal and informal support (eg, in education, healthcare, child services, family), without a corresponding increase in support from adult services.^10^ Many reports being ill-prepared for this transition, during which expectations about their ability to live independently might outweigh their capabilities,^11^ and with uncertainties about where support can be accessed since the move beyond the family and school environments.^12^

It is crucial that autistic individuals receive evidence-based support to improve well-being, and this should be tailored to their needs as autistic individuals transitioning to adulthood. At present, such support is lacking for autistic people of all ages. The 2021 Westminster Commission on Autism report surveyed 585 autistic people and their families in the UK and found that the time following an autism diagnosis was stressful for 78% of respondents and that more tailored information about autism and access to support services would be welcomed, alongside peer-support.^13^ A study about the provision of support to autistic adults without intellectual disabilities identified high variation in services across the country. Postdiagnostic support frequently included support to increase knowledge and understanding of autism, support to develop problemsolving skills and information about services and support. In a UK observational study, autistic individuals who received postdiagnostic support had improved General Health Questionnaire scores and improved daily living outcomes, whereas those who received a diagnosis without support did not see such improvements.^14^ Qualitative evidence and feasibility and pilot studies provide support for the potential of such interventions in young people. Small feasibility studies of internet-delivered autism support for young autistic adults in Sweden showed promise in terms of intervention attendance, acceptability of the intervention and increase in autism knowledge.^15^ A small-scale randomised controlled trial (RCT) of a group-based autism postdiagnostic support programme for autistic young adults in Canada demonstrated improvements in quality of life.^16^ A qualitative evaluation of an autistic-led autism support programme in the UK found positive responses from autistic participants when it was delivered both in person and online.^17 18^ A feasibility study of peer-based support for autistic adolescents and adults in the USA demonstrated promise in terms of participant satisfaction ratings.^19^ Furthermore, a recent systematic review identified that telehealth services show promise in terms of clinical effectiveness and economic impact,^20^ providing support for online intervention delivery.

In adults, such support to understand autism, develop problem-solving skills and increase access to relevant services is typically delivered immediately following diagnosis. However, we know little about support offered to young people, and it may be that such support is required years after a diagnosis. This is particularly likely in cases when the diagnosis was provided at an age when the young person was not able to understand it, or at a time when they were not willing or able to engage with the information. In this paper, we, therefore, define postdiagnostic support as interventions, which are offered at any point after a diagnosis of autism, which aim to help an individual learn about autism, develop strategies to manage daily life and make sense of and accept their autism diagnosis, irrespective of timing with regard to the autism assessment and diagnosis. This definition also fits with adolescent and young adult development, which marks an important period in the negotiation of identity and social relationships.^21 22^ Receiving a diagnosis of autism has significant implications for this process.^23^ There is evidence that having a positive sense of autism identity, autism self-acceptance, and autism community connectedness is associated with better well-being and mental health outcomes when measured quantitatively.^24^ Therefore, autism related support which aims to modify these factors may succeed in improving well-being, mental health and quality of life, and this may be particularly effective when delivered to those who are transitioning to adulthood.

There have been growing calls for more community-led and coproduced supports and services, and for greater autistic involvement in research and practice.^25^ Postdiagnostic support that is codesigned by members of the autistic community is, therefore, more likely to be perceived as credible and acceptable to those who will be accessing it.

The above evidence suggests the potential of a codesigned online group intervention, which promotes autism-related knowledge, social connection and social support within the autistic community. We have codesigned an intervention called *Understanding You, Discovering You* (UYDY) with the autistic community. To develop evidence-based interventions, an RCT is needed, and the first step is to understand the feasibility of running such an RCT.

We are, therefore, conducting a feasibility randomised controlled trial where participants will be randomly allocated eligible and consenting participants at a 1:1 ratio to either UYDY groups and care as usual (CAU). A feasibility design has been selected because this will be the first trial of its kind, testing a new coproduced intervention for autistic young people.

Our objectives were as follows:

1. Assess the rates of recruitment and retention to inform the design of a full-scale RCT.
2. Assess the acceptability of randomisation and outcome measurement procedures to participants.
3. Characterise CAU.
4. Assess the acceptability of the UYDY intervention and CAU.
5. Calculate outcome measure variances for use in a power calculation for a full trial.

### Methods and analysis

### Design

This is the protocol for a two-arm parallel feasibility RCT with a nested qualitative evaluation. We follow here the Standard Protocol Items: Recommendations for Interventional Trials reporting guidelines (see table 1).^26^

**Table 1:** Trial registration information

Primary registry ID number	ISRCTN10513626	
**Date of registration**	5 October 2023	
**Funder**	National Institute for Health Research—Research for Social Care and Autistica (NIHR204276)	
**Sponsor**	University of Bath (UniBath)	
**Contact for all enquiries**	atag@bath.ac.uk	
**Protocol version number**	V1.4	
**Public title**	Autism Transition to Adulthood Group	
**Scientific title**	Peer-group intervention to promote successful transition to adulthood forautistic people: A feasibility randomised controlled trial (RCT) of the Autism Transition to Adulthood Group (ATAG)	
**Countries of recruitment**	England and Wales	
**Interventions**	Understanding You, Discovering You (UYDY) groups Care as usual (CAU)	
**Inclusion criteria**	Age between 16 and 25 years. A clinical diagnosis of autism spectrum disorder (ASD)	
**Exclusion criteria**	Risk of suicide Risk of harm to others Have attended autism psychoeducation or post-diagnostic support with a professional over the past 12 months English, non-English and Welsh literacy levels such that the intervention materials are inaccessible without reasonable adjustments and a supporting person is not available	
**Trial design**	Feasibility randomised controlled trial with two arms, UYDY and CAU	
**Date of first enrolment**	December 2023	
**Sample size**	70	
**Recruitment status**	Recruiting	
	**Objectives**	**Outcome measures**
	Assess the rates of recruitment and retention to inform the design of a full-scale RCT	Warwick-Edinburgh Mental Wellbeing Scale (WEMWBS) Autism social identification measure Health related quality of life (EQ-5D-5L) Interpersonal Support Evaluation List—short UCLA loneliness scale - short Use of services questionnaire based on the Client Service Receipt Inventory Carer measures: WEMWBS and Adult Social Care Outcomes (ASCOT-carer)
	Assess the acceptability of randomisation and outcome measurement procedures to participants	
	Characterise CAU	
	Assess the acceptability of the A-TAG intervention and CAU	
	Calculate outcome measure variances for use in a power calculation for a full trial	
**Ethics review**	Ethical approval for the study granted by the Health Research Authority and Wales REC 5 (Reference: 23/WA/0113)	
**Study duration**	Funding start date: 01 September 2023 Anticipated duration: 22 months (*total; subject to change*) Anticipated end date: 30 June 2025 (*subject to change*)	
**IDP sharing statement**	Participants are asked whether they consent to their anonymised data being shared with other researchers who have received appropriate ethical approval for their research. This will be made available to other eligible researchers on request.	

### Study setting

This trial will be delivered through a partnership between academic institutions, charities (primarily Ambitious and Autism) and autistic individuals and other stakeholders, including health and social care professionals. Recruitment will be conducted via charities, for example, Ambitious about Autism, National Autistic Society and Autistica and lists of potential research participants, for example, at the Centre for Applied Autism Research at the University of Bath. We will also recruit from one pilot NHS autism service as a participant identification centre (PIC) to understand the feasibility of recruitment in NHS settings.

### Eligibility criteria

Inclusion criteria

- Aged 16–25 years at the point of randomisation.
- A clinical diagnosis of Autism Spectrum Disorder is verified by clinical letter or report.

### Exclusion criteria

- Participants who report that they have received any autism postdiagnostic support over the past 12 months will be excluded from the study. This is operationalised as any specific support from a health or social care professional about what an autism diagnosis means, how autism may affect you, and how to find support as an autistic individual. This includes meeting with a professional in an individual or group setting to talk about what autism is. It does not include any self-help through websites or reading, nor does it include support from educational professionals.
- Risk of harm to self that would mean that this intervention is not clinically appropriate, assessed using item 9 of the PHQ-9 and follow-up by a clinician. Where an individual requires intensive mental health support to reduce their level of risk, the individual will not be eligible for this study.
- Risk of harm to others such that group participation would be contraindicated. This will be assessed by asking a series of questions about the individual’s school exclusion and forensic history.
- English, non-English and Welsh literacy levels such that the intervention materials are inaccessible.

### Interventions

#### Understanding You, Discovering You

The intervention is the UYDY groups, which is a peer-group intervention aimed at supporting the transition to adulthood in autistic young people. This multicomponent intervention aims to: (1) provide education about autism, (2) enhance capacity to identify and make use of relevant health and social care services when required and (3) generate affiliation to and social connections within the autistic community.

A process of codesign led by Ambitious about Autism generated a 6-week online peer-group intervention. The process of codesign is documented in depth in a separate paper.^27^

Six online, hour-long, weekly sessions, in groups of 7–10 participants, will be cofacilitated by an autistic person and two professionals from a range of social care and educational backgrounds, for example, social workers, occupational therapists, experienced support workers. The facilitators lead the online sessions, support participation of discussions and take responsibility for the welfare of participants during the sessions. They also facilitate after-session debriefs, complete fidelity forms and attend regular supervisions. The autistic facilitators also role model a positive autistic identity and provide positive affirmation to young people’s experience of autism.

Facilitators will receive monthly supervision of their practice and 7 hours training provided by two qualified clinical psychologists and the ambitious about autism programme manager (KC, WM and BR), which will cover: understanding the needs of autistic young people; current service provision for this group and signposting; adapting communication for autistic young people; techniques in delivering online group interventions.

Following each group session, one facilitator will complete a Facilitator Record Form, which will record which elements of the intervention were completed and any issues identified with the UYDY intervention including observations of participant engagement.

The six UYDY sessions are structured using a facilitator manual and standardised session materials in the format of presentation slides and private online workspaces where participants can share answers to questions and participate in quizzes. The sessions cover topics through didactic and experiential learning. Young people are encouraged to share their experiences and knowledge linked to the topic of each session in structures, and the autistic facilitator also is prompted to share their experiences to provide examples. Topics covered over the 6 weeks include: understanding the autism label; autistic strengths; problemsolving and goal setting; disclosing one’s diagnosis and understanding one’s rights; identifying needs and accessing services when appropriate; identifying and meeting one’s social needs. Participants receive the agenda and session materials for each session by email.

Educational resources will be offered to those nominated by the participant as a supporting person, whether a carer, partner or family member. These are written resources, which map onto the session content delivered to young people. They are sent to carers at the start of the study, and carers are encouraged to read these week-by-week as the young person attends the group sessions.

Participants receiving UYDY will also be able to access CAU, which will be measured for participants in both arms of the trial.

The UYDY Programme Team will contact any individuals who do not attend, to encourage them to attend future sessions.

#### Care as usual

Since this is a pragmatic trial, CAU will continue without restriction, including referrals to health and social care provision for autistic young people. Participants allocated to CAU will be given the opportunity to access the UYDY groups on completion of their final follow-up.

### Outcomes

The main outcomes from the feasibility trial will be collecting data on^1^ recruitment and retention rates^2^; the acceptability of randomisation and outcome measurement procedures^3^; CAU accessed by participants^4^; acceptability of the interventions^5^; clinical outcome measure variances (see below). See the Statistical methods section below for how this will be assessed in the current study.

The primary clinical outcome is the self-report outcome measure Warwick Edinburgh Wellbeing Scale (WEMWBS) at 16-week postrandomisation. Well-being was the measure, which best captured the overall intended outcomes of the multifaceted intervention. This measure is validated for use in this age group and with good internal consistency in autistic adults.^28 29^ This 14-item scale was found to have good internal reliability in a sample of autistic young people aged 15–22 years, and we will assess internal reliability of the measure in 16–25-year olds in this study. Items include ‘I've been feeling confident’, scored on a Likert scale from 1 ‘none of the time’ to 5 ‘all the time’, with higher scores indicating better well-being.

The secondary clinical outcome measures are as follows. The autism social identification measure^30 31^ is a measure of affiliation with the autistic community and identity, with items such as ‘I am glad to be autistic’. This 14-item scale has good reliability and construct validity in autistic adults^32^ and good reliability in autistic young people aged 15–22 years. Quality of life will be measured using the EQ-5D-5, which has been used in previous work with autistic adults.^14 33^ The Interpersonal Support Evaluation List-short^34^ will be used to measure perceived social support, as previously used with autistic adults.^14^ The four-item short UCLA loneliness scale (ULS-4)^35^ will be used to measure experiences of distress due to a discrepancy between actual and desired social connection.

A use of services questionnaire based on the Client Service Receipt Inventory^36^ will be used to characterise CAU by thorough measurement of the additional health and social care services accessed by all participants during the study.

Carer impact will be measured using the ASCOT-carer,^37^ a validated measure of social care-related quality of life for carers, and the WEMWBS.

See table 2 for full details of trial assessments and timepoints.

**Table 2:** Quantitative trial assessments and key participant-related procedure

Data collection timepoint (→)	Prerandomisation		Postrandomisation			
Key quantitative measures and trial procedures for autistic young people	Identification/screening	Baseline	1–8 weeks	8 weeks	16 weeks	24 weeks
Screening	●					
Eligibility assessment	●	●				
Consent to join trial		●				
Receive Understanding You, Discovering You (UYDY) or care as usual (CAU)			Access UYDY or CAU			
UYDY facilitator records forms			●			
Warwick Edinburgh Mental Wellbeing Scale (WEMWBS—primary outcome at 16 weeks)		●		●	●	●
Autism social identification measure		●		●	●	●
Quality of life/utility (EQ-5D-5L)		●		●	●	●
Interpersonal Support Evaluation List—short		●		●	●	●
UCLA loneliness scale		●		●	●	●
Health and social care resource use (Client Service Receipt Inventory)		●		●	●	●
Key quantitative measures and trial procedures for carers						
Consent to join trial and complete outcome measures	◊	◊				
Receive carer educational resources			Access carer educational resources			
Social care-related quality of life for carers (ASCOT-carer)		◊			◊	
WEMWBS		◊			◊	

● Data capture/outcome measures; completion methods may vary depending on participant preferences. ◊ Completed by caregiver.

ASCOTAdult Social Care Outcomes

### Participant timeline

After participants express their interest in the study via an online form, they will be contacted by the research team to conduct a preliminary assessment of their eligibility. If potentially eligible, they will be invited to a baseline assessment. If they meet eligibility criteria and consent to participate, they will become a trial participant. Randomisation will then be conducted within 2 weeks of the next group start date and communicated to the participant by email. Those enrolled in the study will receive updates from the study team such as blog posts. Follow-up assessments will be completed 8, 16 and 24 weeks after randomisation. Participants will be paid with a £15 voucher for each baseline and follow-up assessment they complete.

### Sample size

The recruitment target is 70 participants from across England and Wales. A sample size of 70, with 80% follow-up rates, is deemed sufficient to inform a sample size calculation for a full RCT and to evaluate the rates of recruitment and retention with sufficient precision.^38^

### Recruitment

Autism Transition to Adulthood Group (ATAG) recruitment materials (eg, posters and leaflets) will be shared electronically via charities (eg, Ambitious about Autism, National Autistic Society, Autistica). Recruitment materials will also be shared via social media and traditional media resources (eg, Twitter, websites, press releases) as well as various community organisations including charities and third sector providers of support services.

Potential participants will be identified within the NHS PICs by clinical services staff. This can be done within the context of a clinical appointment or by clinician review of patient lists. Service staff may discuss the study with individuals during a consultation and provide them with an ATAG Study advert.

All potential participants will be referred to complete an ATAG Expression of Interest Form. The form can be completed by potential participants or those who support them via a secure online weblink, or via paper equivalent or on the telephone with a researcher if required. The aim of the expression of interest form is to ask individuals a short series of questions based on the inclusion/exclusion criteria. Those who do not meet the eligibility criteria will be informed of the outcome and no further data about the individual will be collected.

If the individual is potentially eligible to take part, additional information will be requested to allow for further eligibility assessment and participation. Those who are still potentially eligible will be invited to an online baseline assessment, and any accessibility needs or reasonable adjustments will be taken into consideration to allow access to this appointment.

The individual will be sent the baseline questionnaires via an online survey link in advance of this appointment. These appointments will be offered via video call as default, but can also be offered via voice only or phone call. The baseline appointment is structured with an accessible PowerPoint presentation, which potential participants receive in advance, so that they have time to prepare for the appointment. The eligibility criteria will be reviewed, the trial will be explained and the participant can ask any questions. At this point, there will be an assessment of risk to self and others following a risk-standardised operating procedure. This involves completing the Q9 from the PHQ-9 (‘Over the last 2 weeks, how often have you been bothered by thoughts that you would be better off dead or of hurting yourself in some way?’). To assess risk to others, participants will be asked a series of questions developed for this study about risk to others. This will include asking about any incidents of school exclusion or contact with the police due to posing a risk to others. The participant will also be told about the proposed randomisation date and group start dates.

During this baseline discussion, the researcher will check that individuals are able to provide fully informed consent. This will be through asking a series of questions, which check understanding of trial procedures; the right to withdrawal and that their involvement is completely voluntary. For example, to assess their ability to voluntarily consent, they will be asked ‘do you think you have to take part in this research?’.

For individuals who do not meet the eligibility criteria, their baseline questionnaire will be destroyed, and they will be offered a £15 gift voucher to recompense their time. If the researcher believes that the potential participant is fully eligible for the study, they will invite the participant to complete the consent form. Informed consent for both the trial (essential) and nested qualitative study (optional) will be captured via an eConsent (online) form.

If the baseline appointment is more than 2 weeks before randomisation will occur, the WEMWBS (primary outcome) will be sent to participants to complete in the 2 weeks before randomisation.

### Allocation

The randomisation sequence will be generated by Sealed Envelope. Randomisation will be stratified by age (16–17 or 18–25). Participants will be randomised to one of two treatment groups on a 1:1 ratio, that is either UYDY (intervention arm) or CAU (control arm).

The CI (or authorised delegate) will sign into the secure online randomisation system, enter the individual’s unique study ID number and age; they will then receive the code that allocates the participant to the study treatment. The research team will inform the individual of their allocation and securely share the contact details of those allocated to UYDY to the team facilitating the groups. The unblinded randomisation code will be held by selected members of the Trial Management Group (TMG).

### Blinding

The TMG will be blinded to the allocation of treatment group, except for members of staff involved in data management, trial facilitators and supervisors of trial facilitators.

### Data collection methods and data management

Source data for this trial consists of electronic versions of preliminary screening and expression of interest forms, consent form(s), participant and carer completed questionnaires and other records specific to the study. Data from all participants will be collected and retained in accordance with the UK Data Protection Act 2018 and UK General Data Protection Regulation 2018. Participants will be asked to consent to their personal information and research data being stored by the research team.

Standardised outcome instruments will be used throughout the trial; the components and timing of follow-up measures are shown in table 2. All identifiable participant data will be entered into and stored on encrypted databases. All administrative and clinical study data will be stored in separate Questionpro forms. Questionpro is a secure, web-based electronic data capture system designed for the collection of research data. The clinical data will be stored separately to the administrative data. Anonymised clinical data are linked by a study participant ID. Data will be retained for at least 5 years after the end of the trial, and at the end of the archiving period, will be destroyed by confidential means with the exception of a final dataset, which will be made available for data-sharing purposes.

Anonymous research data, including anonymised transcripts, will be stored securely and kept for future analysis with participant consent.

Participant retention will be targeted through sharing coproduced study blog posts to increase engagement.

### Statistical methods

We will follow Consolidated Standards of Reporting Trials (CONSORT) guidelines throughout and present a CONSORT flowchart in the results. We will also report baseline characteristics of participants by arm.

To meet objective 1, assessing recruitment and retention, the percentage of those who are eligible and consent to be randomised will be calculated. Retention rates will be calculated as percentages of the number of participants in each arm, who complete follow-up measures at each time point. All data will be used as randomised, and a percentage of missing data for each scale at each follow-up time point will be reported.

To meet objective 2, about the acceptability of randomisation and outcome measurement procedures. Qualitative data are collected (see below).

For objective 3, CAU will be characterised by calculating the proportion of participants in CAU who receive each type of support.

For objective 4, the acceptability of the interventions will be assessed quantitatively by calculating the percentage of those who complete the intervention, the mean number of sessions completed (with three sessions considered the minimum acceptable number), and the SD as well as the number of adverse events (AEs) in each arm.

For objective 5, outcome measure variances will be assessed with descriptive statistics, including means and SD, calculated by intervention group and timepoint for each outcome measure. This will inform the power calculation for a fully powered trial.

### Carer impact substudy

The carer data will also be analysed as described in objectives 1 and 5 above.

### Qualitative study

We will conduct a qualitative study to meet aims 2 and 4, regarding the acceptability of randomisation and outcome measurement procedures and interventions (UYDY and CAU). Qualitative work with those who drop out of the study and those who continue attending will help us understand the factors contributing to any treatment drop-out.

All aspects of this part of the study (ie, design, recruitment, interviewing, analysis, interpretation of findings, dissemination) will be conducted in partnership with autistic young people who are in the leadership team for the study.

All participants in the trial will be asked if they are willing to be contacted about taking part in an interview at the time of trial consent. Verbal consent will be taken at the point of interview for autistic young people and carers. Facilitators will complete written and audio consent at the point of interview.

Semistructured one-to-one interviews will be conducted with 20 participants allocated to UYDY, 10 participants in CAU, 10 group facilitators and 10 carers of participants. Purposive sampling will be undertaken, aiming for diversity of qualitative study participants with respect to ethnicity, gender, age, and geographical region, to ensure a range of viewpoints.

Interviews will be conducted online via video or audio-only calls. The topic guide will be developed with autistic young people and will cover areas including:

- Experiences of being recruited into the study.
- Equipoise: whether participants had a preference for allocation to CAU or UYDY.
- Experience of the interventions and follow-up assessments.
- Suggestions for improvements to UYDY.
- Suggestions on how to increase access and inclusion for minoritised participants (where applicable).

With informed consent, interviews will be recorded using an encrypted audio recorder for telephone interviews or the secure university-approved online platform MS Teams or Zoom. Interviews with autistic participants will be conducted by an autistic young person wherever possible, and autism adaptations will be made depending on individual participant need. Adaptations will include to communication method (eg, using the chat function where the individual is struggling to verbally communicate), environment (eg, ensuring the individual has access to fidget toys where needed) and structure (eg, having regular breaks where needed). Qualitative data collection and analysis will be conducted concurrently, with the topic guide being updated based on early interviews.

### Qualitative analysis

Interview recordings will be fully transcribed, anonymised, checked for accuracy and imported into NVivo qualitative data analysis software to aid data management.

Qualitative data will be analysed using Reflexive Thematic Analysis from a critical realist standpoint, as defined by Braun and Clarke.^39^ Analysis will begin shortly after data collection starts, will be ongoing and iterative. Analysis will inform further data collection: for instance, analytic insights from data gathered in earlier interviews will help identify any changes that need to be made to the topic guide during later interviews.

The neurodiverse qualitative team will work together to conduct line-by-line coding of the transcripts and theme development to generate a robust analysis. A subset of transcripts will be analysed collaboratively in group meetings to ensure that a consistent approach to data analysis is undertaken. The team analysis will then be presented to the other coapplicants and stakeholder groups to ensure that the findings are understood as plausible, rigorous and transferable.

### Patient and public involvement

There has been significant patient and public involvement at all stages of this project. We are a neurodiverse team including autistic young people, researchers and professionals. We have consulted various stakeholders in the design of the research and dissemination plans, including autistic young people, a stakeholder group of professionals and parents, and a network of social care professionals. The intervention itself was also codesigned in a partnership between ambitious about autism and academic partners.^27^

### Risk and adverse events

A risk management standardised operating procedure has been developed for use by both research and social care staff throughout the trial. This will be implemented to ensure the safety of all participants if they express significant distress during the research process or a desire or intention to harm themselves or others.

AEs are expected throughout the course of this trial and will be recorded by the research team. These will be detected via study questionnaires and follow-up by the research team as well as contact with the group facilitators for those allocated to UYDY. The research team will categorise whether AEs are serious, expected and related to trial participation. Serious AEs are those that are life threatening or result in death, require inpatient hospitalisation or prolonging existing hospitalisation or result in persistent or significant disability or incapacity. Only non-serious AEs deemed to be linked to trial participation will be recorded. All serious AEs, which are related to trial participation, will be recorded and reported to the sponsor and Trial Steering Committee chair within 24 hours of the study team becoming aware of them.

### Ethics and dissemination

Ethical approval for the study was granted by the Health Research Authority and Wales REC 5 (Reference: 23/WA/0113). The trial was designed independently of the trial sponsor and funder. The trial will be monitored and audited in accordance with the Sponsor’s policy, which is consistent with the UK Policy Framework for Health and Social Care Research. Modifications to the trial procedures will be reported to all relevant parties and appropriate ethical approval will be sought for such changes. Fully informed consent will be gained from all participants; to ensure that individuals have read and understood the information sheet, researchers will ask them to summarise their understanding of trial procedures, the voluntary nature of participation and right to withdraw, before the consent form is presented (see online supplemental material).

The combined Data Monitoring and Trial Steering Committee is independent of the sponsor and no competing interests have been reported. It includes experts in child mental health trials, autism intervention and research, and an independent statistician.

#### Dissemination

An engagement plan will be produced with the TMG, collaborators and members of the Patient Advisory Group. We will seek input from the Trial Steering Committee about the suitability of engagement plans and the dissemination policy.

In terms of the overall study findings, we will produce papers in peer-reviewed journals, presentations and blogs, sharing findings both in academic settings (conferences) and with the autism community (stakeholder events) as well as policymakers and professionals. Our results will inform the design of a full RCT. Authors of all publications will meet the ICMJE criteria for authorship.
