# Supplementary material for: Autism Transition to Adulthood Groups (ATAG): protocol for a feasibility RCT of a new peer-group intervention to promote successful transition to adulthood for autistic young people
Source: BMJ Open. 2024 Nov 19;14(11):e089802. doi: 10.1136/bmjopen-2024-089802 (PMC11580241; doi:10.1136/bmjopen-2024-089802)
Supplement: Supplementary file 1 [file bmjopen-2024-089802.reviewer_comments.pdf]

PEER REVIEW HISTORY

BMJ Open publishes all reviews undertaken for accepted manuscripts. Reviewers are asked to complete a checklist review form and are provided with free text boxes to elaborate on their assessment. These free text comments are reproduced below.

ARTICLE DETAILS

Title (Provisional)

Autism Transition to Adulthood Groups (ATAG): protocol for a randomised controlled feasibility trial of a new peer-group intervention to promote successful transition to adulthood for autistic young people

Authors

Cooper, Kate; Burnley, Annabel; Allain, Leon; Beresford, Bryony; Crane, Laura; Vazquez Morales, Maximiliano; Portway, Lucy; Redmayne, Benjamin; Russell, Ailsa; Mandy, W

VERSION 1 - AUTHOR RESPONSE

1) Please include the clinical trial registry name and number in the Trial Registration section of the submission system.

DONE

2) Aside from the marked copy, please also provide a clean copy of your manuscript, without 'tracked changes' or highlighted text, and upload it in ScholarOne under the file designation 'Main document'.

DONE

3) Please ensure that author affiliations (including departments) are identical on both the Title Page and submission system. Any secondary affiliations must also be listed in both.

I have done my best, but really struggled with the online author information system so it's not completely identical.

4) Please ensure that all authors are included in your author contributor statement and that it is identical in the manuscript and submission system. For further information please see https://authors.bmj.com/policies/bmj-policy-on-authorship/

DONE

5) We note that copyright brands are mentioned in the manuscript but the relevant permissions to use these have not been provided. Please upload these permissions or remove the branding.

I don't believe copyright is mentioned in the paper?

*Please update the article title to specify that the present paper is a protocol, to avoid the abbreviation RCT. Eg, "Autism Transition to Adulthood Groups (ATAG): protocol for a randomised controlled feasibility trial of a new peer-group intervention to promote successful transition to adulthood for autistic young people" (or similar).

Thank you for this very helpful suggestion – we have changed the title as suggested.

*Please move the 'Strengths and limitations of this study' section to after the abstract and please add one or two additional bullet points highlighting the key limitations of the study.

Thank you for highlighting that we had not listed a single limitation – this has now been amended.

*We note that the main objectives of the study include assessment of recruitment, retention, acceptability, etc, as is appropriate for a feasibility study. However, we found it strange that none of these measures are defined as study outcomes and that the study primary and secondary outcomes are all clinical measures/efficacy-like outcomes. Can you clarify the reason for this?

This section has now been updated to clarify that the outcomes are indeed linked to the feasibility of a full RCT.

*Relatedly, it would be useful to update the abstract 'Methods and analysis' sections to include mention of the assessment of these feasibility outcomes before mentioning the 'primary' and 'secondary' (clinical) outcomes.

This has been updated as you suggest.

*Please further update the 'Ethics and dissemination' section of the abstract to include a reference number(s) for the ethics approval(s), if available.

This has been added.

*Please add a fourth section at the end of the abstract as follows: Trial registration: ISRCTN10513626.

This has been added.

*The information on adverse events monitoring currently in the 'Ethics and dissemination' section should be moved to an appropriate place in the 'Methods and analysis', since this is part of the study methods. This applies to both the abstract and the main text.

These have now been moved to the end of the methods section and amended in the abstract

*Please further update the 'Ethics and dissemination' section of the main text to include brief mention of participant informed consent requirements

This has been added.

*Additionally, please update the 'Dissemination' subsection to mention that results will inform the design of a full RCT and will be disseminated through peer-reviewed journals, conferences, and stakeholder events, consistent with the statement in the abstract.

This has been added.

*Please make the ‘Supplementary information’ table at the end of the manuscript into a numbered table, cited in the main text, or a supplemental file (appendix), cited in the main text. If moving to an appendix, please ensure that key information in this table (including the date on which recruitment began and the planned or expected timeline for completion of the study) are also included in an appropriate place in the main text.

We have opted to include this as table 2 which is referenced in the design section.

*Along with your revised manuscript, please provide an example of the participant consent form as a ‘Supplemental Material’ file (appendix), as per item #32 of the SPIRIT checklist. This file should be cited in the main text where the informed consent procedures are described.

Please find attached in our resubmission

*For all trials that started after January 2019, we require that a data sharing plan is included in the clinical trial registry. This appears to be missing from the registry page for your study (https://www.isrctn.com/ISRCTN10513626): while the registry record indicates ‘No’ under ‘Individual participant data (IPD) Intention to share’, the subsequent sections indicate ‘The current data sharing plans for this study are unknown and will be available at a later date’ and ‘Data sharing statement to be made available at a later date’, which contravenes our requirement that a plan be in place. Please update the registry page to indicate your IPD sharing plan for the study.

I have requested this update on ISRCTN so that it now matched the information provided in the protocol paper submitted to BMJ Open.
